# Objective and Subjective Evaluation of Canal Ledge Management in Dental Students: Educational Performance and Perception Study

**DOI:** 10.7759/cureus.117944

**Published:** 2026-10-14

**Authors:** Eqbal Hasan, Hassan Achour

**Affiliations:** 1 Department of Endodontic and Operative Dentistry, Damascus University, Damascus, SYR

**Keywords:** clinical decision-making, dental education, ledge management, objective performance, procedural skills, subjective perception

## Abstract

Aim: This study aimed to evaluate the objective performance and subjective perception of two endodontic techniques for canal ledge management among dental students and to assess their learning experience and future clinical intentions.

Methods: Dental students with prior mechanical instrumentation experience participated in a controlled experimental setting. Students performed canal ledge management using the T-Mode and manual insertion techniques under standardized conditions. Outcomes included procedural time, perceived difficulty, need for supervision, perceived performance, and preference. Statistical analysis was performed using paired t-tests or Wilcoxon signed-rank tests, Chi-square tests, Fisher’s exact test, McNemar test, Cohen’s kappa, and effect size analysis.

Results: The T-Mode technique demonstrated significantly shorter operating time compared to the manual insertion technique (p < 0.05). No significant differences were observed in perceived learning difficulty, application difficulty, or need for supervision between techniques. While most students correctly identified the faster technique at the group level, individual agreement with actual performance was weak. Preference and perceived outcome quality showed no statistically significant differences, although a tendency toward the T-Mode was observed. Most students reported willingness to adopt T-Mode alone or in a combined approach in future clinical practice. Although objective performance favored the automated technique, subjective evaluations showed limited concordance with measured outcomes.

Conclusion: Students demonstrated flexible, context-dependent clinical decision-making and a strong inclination toward integrating new techniques into future practice. These findings highlight the value of combining objective and subjective assessments in endodontic education and suggest a potential role for structured early exposure to contemporary techniques.

## Introduction

Managing iatrogenic errors is essential in endodontic treatment, particularly in retreatment cases, as such procedural complications may compromise the primary objectives of root canal therapy, including adequate cleaning, shaping, and three-dimensional obturation of the root canal system. Although endodontists are trained to manage these complex situations, less experienced practitioners may treat such cases in clinical practice rather than referring them to specialists. With advances in endodontic knowledge, materials, and instruments, general practitioners may undertake such procedures independently rather than referring them to specialists [1-3]. Therefore, early training in the management of common procedural errors is crucial for improving clinical competence and decision-making in practice, particularly for frequently encountered complications such as canal ledge formation.

Among iatrogenic complications, ledge formation is considered one of the most common procedural errors during root canal treatment [4]. It is defined as an artificial irregularity within the canal that prevents the proper advancement of instruments toward the apical region [5] and is commonly associated with the use of rigid non-precurved instruments under excessive pressure, inadequate access cavity preparation and canal flaring, absence of a glide path, and inaccurate working length determination [6].

The conventional approach to ledge management involves using small hand instruments, with the tip of each file pre-bent before insertion. Pecking motions are applied until the file enters the original canal path, followed by short up-and-down movements while the instrument remains apical to the ledge. This sequence is continued using progressively larger files to gradually eliminate the ledge [7].

Another method for ledge management utilizes nickel-titanium (NiTi) rotary instruments. Following glide path preparation, a pre-curved rotary file is carefully advanced manually until it passes the ledge. The file is then operated by an endo motor, applying controlled short vertical movements until the original working length is regained and the ledge is resolved [8].

An alternative approach is T-Mode protocol incorporated in the Ai Endo Motor (Woodpecker, China). This technique employs a precurved file that is initially advanced using a Safety Glide Path (SGP) motion of 90° CW/90° CCW at 100 rpm to bypass the ledge, representing phase 1 of the protocol (bypassing phase). Once bypass is achieved, the motion is switched, without withdrawing the file from the original path, to a more effective setting to smooth the ledge, defining phase 2 (shaping phase). The reciprocal movement in phase 2 is set at 380° CW/50° CCW with 150 rpm and 3 N.cm torque, as recommended by the manufacturer [9].

A specific nickel-titanium file system designed for ledge management has been introduced by Fanta Dental (Fanta AF™ Ledge Correction (LC)). These files are manufactured from a controlled memory alloy and feature a specialized inactive, pre-bent tip intended for the preparation of canals exhibiting ledges. Previous research has demonstrated superior efficiency of this system in terms of reduced preparation time and improved canal centering during ledge management [8].

Undergraduate endodontic curricula have traditionally focused on teaching the conventional manual approach using hand files for ledge management. However, recent evidence suggests that incorporating engine-driven techniques enhances undergraduate students’ performance and leads to improved clinical outcomes [10]. In addition, these techniques have been shown to improve procedural efficiency and reduce treatment time in both routine canal preparation [11-13] and ledge correction procedures [8].

Despite this advancement, undergraduate students typically have limited exposure to procedural complications, such as canal ledge formation, which may be encountered during primary treatments or retreatment cases. This limited experience can affect their ability to manage such situations effectively in clinical practice. Therefore, integrating structured training in contemporary, evidence-based techniques is essential to bridge this gap and enhance clinical competence.

Accordingly, this study aimed to compare two engine-driven approaches for canal ledge correction under controlled educational conditions. By allowing students to perform both techniques and provide structured feedback on efficiency, ease of use, and perceived effectiveness, the study generates comparative data that integrate objective performance measures with subjective evaluation. This approach contributes to the limited evidence on contemporary ledge management techniques at the undergraduate level and provides insights that may support the evidence-based integration of advanced endodontic procedures into preclinical and clinical training.

## Materials and methods

Participant inclusion criteria

Twenty-one undergraduate students from Damascus University volunteered to participate in the study. An online questionnaire was used to assess eligibility and obtain informed consent before participation. Eligible students had completed two semesters of clinical endodontics and had prior experience with various engine-driven instrumentation systems, including familiarity with their basic concepts and mechanics. Students were excluded if they had not completed basic clinical training in endodontics, had received advanced specialized training in ledge management, had previously participated in similar ledge management experiments using resin blocks, or had received clinical training outside the standard undergraduate curriculum. The final sample size reflected the number of eligible volunteers available during the study period and the practical resources required for conducting the study. The study was conducted from October 8, 2025, to October 22, 2025.

Study design and experimental framework

Each student was interviewed and trained individually to prevent their learning or judgment from being influenced by peers. This individual approach provided a calm and private environment, allowing students to explore the techniques freely, ask questions without hesitation, and fully understand the procedures without feeling self-conscious or pressured.

Initially, each participant completed a form to confirm eligibility and provide information regarding any prior experience managing ledges (Appendix A). At the beginning of the session, a brief background introduction was delivered, covering ledge formation from definition to management protocols. Students were then familiarized with the materials and devices to be used in the experiment.

All participants received standardized, individual instruction before performing each instrumentation technique. The same core information and procedural steps were explained to every participant, and each technique was introduced immediately before its application to avoid overwhelming participants with information about both techniques simultaneously. Participants were encouraged to ask questions, and additional explanations were provided as needed until each participant demonstrated an adequate understanding of the procedure and felt ready to begin. Although the instructional content and approach were standardized, the duration of training was not predetermined and varied according to each participant’s need for clarification. The same instructional approach was followed for both techniques.

Each student performed ledge management using two different engine-driven systems. All participants performed the manual bypass rotary technique first, followed by T-Mode. The order was kept constant across participants to maintain a consistent experimental sequence. Participants were informed that the investigator had no affiliation with or preference for either technique and were encouraged to provide unbiased evaluations based solely on their experience. Before starting each system, the protocol and operational steps were explained thoroughly, and any questions were addressed. Students then applied the method on resin blocks containing pre-prepared ledges; the blocks were mounted vertically in a vise to limit visual access to the canal path and the ledge. The researcher did not intervene during the procedure unless a student requested guidance or clarification. The time required to complete each task was recorded. After completing the first system, students completed a questionnaire developed by the authors to document their initial experience with the technique (Appendix B).

The instructor then introduced and demonstrated the second system in the same manner. Students applied it to another prepared ledged resin block, following the same procedure as for the first system (Appendix C). At the end of the session, students completed the comparative section of the same questionnaire, evaluating the two systems based on their hands-on experience (Appendix D and Appendix E). The questionnaire was developed by the authors specifically for this study. The complete questionnaire, including all questions, response options, and scoring procedures, is provided in the Appendices.

The primary outcome was procedural time, measured objectively for each instrumentation technique. Secondary outcomes included participants’ subjective evaluations of the two techniques, including perceived ease of learning, ease of application, and overall preference, as assessed using the structured questionnaire.

Materials and tools

The materials used in this study included resin blocks with simulated root canals; all canals had a curvature between 25° and 30° and pre-formed ledges. Glide path establishment and recapitulation were performed using size 10 stainless steel K-files (Mani Inc., Tochigi, Japan). Two engine-driven NiTi instrumentation protocols for ledge management were evaluated; their design characteristics and operating protocols are described in a subsequent section. An endodontic motor with adjustable rotational and reciprocating motions (Ai Endo Motor, Woodpecker, China) was used. Both protocols employed Fanta AF™ (CL) NiTi files. Standard endodontic accessories, including rubber stoppers, endodontic rulers, and irrigation syringes, were used throughout the procedures. The time required to complete each ledge management procedure was recorded using a digital stopwatch. Students’ perceptions were collected using structured questionnaires assessing ease of learning, ease of application, and overall preferences.

Preparation and standardization of simulated ledges

The location at which the simulated canal began to deviate from its straight longitudinal axis was identified as the target site for ledge formation. An Exacto bur No. 1 (Angelus, REF 1911, Brazil) was used with intermittent insertion movements to prepare the canal to a point 1 mm short of the predetermined ledge location. A size 35 stainless steel K-file was then directed toward the outer canal wall and advanced under pressure to the predetermined depth, thereby extending the preparation 1 mm beyond the depth reached by the bur.

Subsequently, a Super Files Gold F3 rotary file (Flydent, Shenzhen Flydent Medical Co., Ltd., Shenzhen, China) was introduced into the prepared region and used with brushing movements directed toward the outer canal wall to create a smoother, continuous pathway between the bur-prepared and file-prepared portions of the canal. Distilled water was used to irrigate the canals between successive preparation steps. Following ledge formation, canal patency was checked using a size 8 stainless steel K-file.

All ledges were prepared under a DOM3000 operating microscope (Semorr Medical Technologies Co., Ltd., Suzhou, China) to facilitate visualization and precise control. After preparation, a straight, unprecurved size 10 K-file was inserted into each canal to assess ledge formation. Canals in which the file encountered resistance and could not negotiate the ledge were retained, whereas specimens in which the file passed beyond the ledge into the original canal path were excluded. Each specimen was subsequently examined under the same microscope at ×20 magnification to verify the presence of a ledge at the predetermined location. Specimens exhibiting excessive enlargement, irregular ledge morphology, or other deviations from the standardized preparation criteria were excluded and replaced with resin blocks prepared using the same protocol. The prepared canals had similar characteristics, with canal curvatures ranging from 25° to 30°. For each instrumentation system, participants randomly selected a resin block containing a previously prepared ledge from the available specimens.

Ledge management protocols

Protocol A (Manual Bypass Rotary Protocol)

Initial ledge bypass and glide path creation were performed using a size 10 K-file. After negotiating the ledge manually, Fanta AF™ (CL) NiTi files (Fanta Dental, Shanghai, China) were introduced. Each precurved file was gently advanced past the ledge before being connected to the Ai Endo Motor (Woodpecker, China) for engine-driven instrumentation. The sequence of files used included 10/06, 10/08, 15/06, 20/07, 25/08, and 30/09, operated in a reciprocating motion at 150 rpm with a torque of 3 N.cm (90° clockwise, 30° counterclockwise). Advancement was carried out using controlled in-and-out strokes of 1-2 mm, while anticurvature pressure was applied to facilitate ledge reduction. Irrigation and recapitulation were performed with a size 10 K-file after each file.

Protocol B (T-Mode)

Initial ledge bypass and glide path creation were performed using a size 10 K-file. Canal preparation was performed with the Fanta AF™ (CL) system (Fanta Dental, Shanghai, China). The file sequence matched that of Protocol A: 10/06, 10/08, 15/06, 20/07, 25/08, and 30/09. Each precurved file was attached to the Ai Endo Motor (Woodpecker, China) and operated in T-Mode. During the initial Safety Glide Path (SGP) phase, reciprocal motion of 90° clockwise and 90° counterclockwise at 100 rpm was used until the ledge was bypassed. Once bypassed, the file tip remained apical to the ledge, and the motor was switched to the shaping phase, using reciprocal motion of 380° clockwise and 50° counterclockwise, with a torque of 3 N.cm at 150 rpm. Controlled push-pull strokes of 1-2 mm were applied until reaching full working length, with anticurvature pressure to aid in ledge reduction. Irrigation and recapitulation were performed with a size 10 K-file after each file.

Procedural time measurement

Procedural time was measured using a digital stopwatch, starting when the participant introduced the first file into the canal, after the explanation and demonstration of the technique had been completed and all questions had been addressed. Timing continued until completion of the procedure following the final file, including the final irrigation and patency check. The recorded time included all procedural steps, including file changes, irrigation, working length determination, patency checks between successive files, and any troubleshooting or additional guidance required during the procedure. Time spent on these activities was included because the study aimed to assess the overall time required by participants to perform each technique under the study conditions.

Statistical analysis

Data were analyzed using descriptive statistics (frequencies, percentages, means, and standard deviations (SD)). Normality was assessed using the Shapiro-Wilk test. Depending on data distribution, the Wilcoxon signed-rank test or paired t-test was applied for within-group comparisons. Chi-square tests were used for categorical variables, while Fisher’s exact and McNemar tests assessed associations and paired proportions. Effect size was calculated using Cohen’s d, and agreement between perceived and actual performance was assessed using Cohen’s kappa. Statistical significance was set at p < 0.05. Statistical analyses were performed using IBM SPSS Statistics for Windows version 26.0 (IBM Corp., Armonk, NY).

## Results

Demographic data and previous experience (pre-experiment)

All 21 students had prior experience with automated preparation systems (100%). A total of 17 (81%) students reported having encountered ledge formation during their previous endodontic treatments, whereas 4 (19%) had not encountered this problem previously. When asked about their preferred approach to managing a ledge within the root canal, 14 (66.7%) preferred a combination of hand and automated files, making it the most commonly selected approach, while 6 (28.6%) preferred bypassing the ledge using hand files alone. Only 1 (4.8%) student preferred not to bypass the ledge and to leave it unchanged.

Relationship between the presence or absence of prior experience in dealing with the problem of ledge formation and preferred management strategies (pre-experiment)

Students were divided into two groups, those who had encountered the problem and those who had not, to identify their preferred management strategies within each group.

Students with actual experience of the problem showed a preference for the hybrid approach (manual + automated files), which was selected by 11 (64.7%) of them. This was followed by the manual-only approach in 5 (29.4%) cases, while leaving the ledge unbypassed was selected by 1 (5.9%).

Among students without prior exposure to the problem, 3 (75%) also selected the hybrid approach, whereas 1 (25%) opted for the manual-only method. None of the students in this group chose to leave the ledge unbypassed.

The Fisher-Freeman-Halton exact test was used to examine the relationship between prior experience with the ledge problem and the preferred management approach. The test yielded a value of 0.693 with an exact two-sided p-value of 1.000. The results showed no statistically significant association between previous experience and preference for handling method.

Comparison of learning difficulty and application difficulty between the two techniques

The Shapiro-Wilk test results indicated a statistically significant deviation from normality; therefore, non-parametric tests were applied in the subsequent analysis.

The descriptive statistics indicate that students’ evaluations of both learning and application difficulty for Technique 1 (manual insertion) and Technique 2 (T-Mode) were relatively comparable. The mean learning difficulty was 1.95 for both techniques, with a higher standard deviation observed in Technique 2, representing greater variability in students’ responses. Regarding application difficulty, it was slightly higher than learning difficulty in both techniques, with a mean of 2.19 for Technique 1 and 2.05 for Technique 2. The minimum and maximum values show that all responses remained within a low to moderate range (Table 1).

**Table 1:** Statistics of the difficulty of learning and application Difficulty scores: 1 = very easy, 5 = very hard

Technique	Variable	Number	Mean	Standard deviation	Minimum	Maximum
Technique 1 (manual insertion)	Difficulty of learning	21	1.95	0.669	1	3
	Difficulty of application	21	2.19	0.814	1	4
Technique 2 (T-Mode)	Difficulty of learning	21	1.95	1.071	1	4
	Difficulty of application	21	2.05	0.921	1	4

Wilcoxon Signed-Rank Test Results

The Wilcoxon signed-rank test was used to compare learning and application difficulty between the two techniques, as well as within each technique. The test indicated that perceived difficulty did not differ significantly either between the two techniques or within each technique across learning and application stages (Table 2).

**Table 2:** Wilcoxon signed-rank test statistics Note: Technique 1: manual insertion, Technique 2: T-Mode

Comparison	Z-value	p-value (two-tailed)
Difficulty of learning: Technique 1 versus Technique 2	0.000	1.000
Difficulty of application: Technique 1 versus Technique 2	-0.731	0.465
Learning versus application (Technique 1)	-1.508	0.132
Learning versus application (Technique 2)	-0.632	0.527

Time required for performing each technique

Normality was confirmed prior to applying the paired samples t-test. The T-Mode technique was significantly faster than the manual insertion technique, with a mean difference of approximately 114 seconds (~1.9 minutes) (p = 0.019, Cohen’s d = 0.558). This was consistent with descriptive results, where T-Mode showed a lower mean time (716.33 versus 830.33 seconds), similar variability between groups, and a slightly narrower time range (Table 3).

**Table 3:** Statistics of the required time df: degrees of freedom

Descriptive statistics of required time (in minutes)					
Technique	Median		Mean ± SD	Range (min-max)	
Manual insertion technique	13.28		13.84 ± 2.75	9.03-19.67	
T-Mode technique	11.93		11.94 ± 2.74	7.85-16.42	
Comparison of time between the two techniques (paired samples t-test)					
Parameter		Value			Interpretation
Mean difference		144 seconds (~1.9 minutes)			T-Mode faster
t-value		2.559			Greater than critical value
df		20			-
p-value		0.019			Statistically significant < 0.05
95% confidence interval		21.08-206.92			Does not include zero (significant)
Effect size (Cohen’s d)		0.558			Moderate effect

Perceived faster technique (from students’ perspective)

The Chi-square goodness-of-fit test revealed a highly significant difference in the distribution of students’ perceptions of the faster technique (χ² = 17.429, df = 2, p < 0.001). The majority of students (16 (76.2%)) perceived the T-Mode technique as the fastest, while a smaller proportion (3 (14.3%)) considered the manual insertion technique to be faster, and 2 (9.5%) reported no difference.

Agreement between actual time performance and students’ perception of the faster technique

Overall, 13 (61.9%) students correctly identified the faster technique, while 8 (38.1%) students showed a mismatch between perceived and actual performance. Correct identification was higher among students for whom the T-Mode technique was actually faster (11 (84.6%)) compared to those for whom the manual insertion technique was faster (2 (25%)).

A Fisher-Freeman-Halton exact test showed no statistically significant association between actual performance and students’ perceptions (p = 0.595). The overall agreement rate was 13 (61.9%), with a weak level of agreement (Kappa = 0.196), indicating limited concordance between perceived and actual performance.

Need for supervisor assistance during application

A lower proportion of students required supervision with the T-Mode technique (8 (38.1%)) compared to the manual insertion technique (13 (61.9%)). However, the McNemar test showed no statistically significant difference between the two techniques (p = 0.625), indicating comparable supervision requirements. At the individual level, most students either required supervision in both techniques (10 (47.6%)) or in neither (7 (33.3%)). Only a small proportion showed differences between techniques, with 3 (14.3%) students requiring assistance in the first method and 1 (4.8%) student in the T-Mode technique only. Despite this imbalance, the difference did not reach statistical significance.

Analysis of preferences and overall evaluation of the two techniques

After completing both techniques, students were asked to evaluate their overall preferences, including the technique they preferred, the one considered to produce better results, and their intention to use these techniques in future clinical practice. These variables were analyzed using descriptive statistics. A Chi-square goodness-of-fit test was conducted to evaluate whether the observed distribution of preferences significantly differed from an assumed uniform (equal) distribution across both techniques.

Preferred technique (from students’ perspective)

The results showed that the T-Mode technique was the most preferred among students, with 14 (66.7%) students selecting it compared to 7 (33.3%) students who preferred the manual insertion technique. However, this difference did not reach statistical significance (χ² = 2.333, df = 1, p = 0.127).

Perceived outcome quality (from students’ perspective)

When students were asked to evaluate which technique produced better results, 8 (38.1%) reported that the T-Mode technique was superior, while an equal proportion (8 (38.1%)) reported no difference between the two techniques. In contrast, 5 (23.8%) considered the manual insertion technique to yield better results.

A Chi-square goodness-of-fit test showed no statistically significant differences between the response categories (χ² = 1.429, df = 2, p = 0.489).

Intended future clinical use of the techniques

Students were asked which technique(s) they intend to use in future clinical practice when encountering similar cases of canal ledges. Most students (9 (42.9%)) reported that they would rely solely on the T-Mode technique, while 8 (38.1%) stated that they would use multiple techniques depending on the clinical situation. In contrast, 3 (14.3%) preferred using the manual insertion technique only, and 1 (4.8%) reported a hybrid approach starting with manual preparation for larger sizes using hand files before switching to automated instruments.

Overall, 17 (81%) students indicated that they would include the T-Mode technique in their future clinical practice, either as a standalone method or as part of a combined strategy.

Assessment of scientific novelty

Most students (12 (57.1%)) reported that both techniques were new to them, while 8 (38.1%) indicated that only the T-Mode technique was new. In contrast, only 1 (4.8%) student reported prior familiarity with both techniques. Overall, 20 (95.2%) participants had no prior knowledge of both methods before the experiment.

Students’ evaluation of the scientific benefit of the experiment

All students (21 (100%)) reported that participation in the experiment provided them with scientific benefit.

Students’ evaluation of the adequacy of pre-instruction for both techniques

All students (21 (100%)) reported that the pre-instruction provided for both techniques was sufficient to clarify all aspects of the procedures.

Analysis of open-ended questions

Students’ Comments Regarding Technique 1

The most frequently reported issue was difficulty in attaching the file to the handpiece inside the canal (5 (23.8%)). Overall, however, a considerable proportion of students reported no specific concerns, with generally positive feedback (Table 4).

**Table 4:** Students’ comments regarding each technique Students could provide more than one comment; therefore, the total number of responses exceeds the number of participants and percentages may not sum to 100%.

Students’ comments regarding the manual insertion method		
Category	Number (%)	
No comments	9 (42.9%)	
Difficulty in attaching the file to the handpiece inside the canal	5 (23.8%)	
General positive feedback (comfortable, effective, fast)	3 (14.3%)	
Caution regarding fracture or over-preparation	2 (9.5%)	
Prior experience with the technique	2 (9.5%)	
Time and effort consumption	1 (4.8%)	
Students’ comments regarding the T-Mode method		
Category		Number (%)
No comments		11 (52.4%)
Faster than Technique 1		6 (28.6%)
Safer than Technique 1		4 (19%)
More comfortable and provides better control		3 (14.3%)
Difficulty in guiding the file in curved canals		2 (9.5%)
Effective in bypassing ledges in molars		1 (4.8%)

Students’ Comments Regarding Technique 2

Overall, the T-Mode technique was most commonly perceived as faster (6 (28.6%)) and safer (4 (19%)) compared to the manual insertion technique. In addition, more comfortable handling and improved control were reported by some students. More than half of the participants (11 (52.4%)) reported no specific comments (Table 4).

Clarification of Students Who Selected “Multiple Techniques” (n = 8)

When asked about their intended future clinical approach after completing the experiment, a subgroup of students reported using more than one technique depending on the clinical situation. Overall, those who preferred a combined strategy described diverse, case-dependent decision-making influenced by anatomical factors, technical difficulties, availability of equipment, and instrument quality, reflecting a flexible and context-based clinical approach (Table 5).

**Table 5:** Students’ comments regarding using combined approaches

Strategy	Number (%)
Depends on case (location of ledge, severity)	1 (12.5%)
T-Mode for molars, manual insertion for anterior teeth	1 (12.5%)
Use T-Mode when insertion is difficult	1 (12.5%)
Start with conventional hand files, then switch to T-Mode	1 (12.5%)
Start with T-Mode then switch to conventional method	1 (12.5%)
Manual insertion when the device required for T-Mode is unavailable	1 (12.5%)
Manual insertion when canal path is difficult to identify	1 (12.5%)
T-Mode requires high-quality files	1 (12.5%)

Overall findings from open-ended questions

Overall, open-ended responses indicated that the main limitation of the manual insertion technique was technical difficulty during file attachment, whereas the T-Mode technique was primarily associated with speed, safety, and higher acceptance. T-Mode was more frequently preferred (6 (28.6%)) compared to the manual insertion technique (4 (19%)) and was also commonly perceived as faster. However, the manual insertion technique remained valued for its superior tactile feedback and perceived safety. A combined approach was accepted by a proportion of students (4 (19%)).

Suggestions for improving the experiment

Overall, students expressed a very high level of satisfaction with the experiment, with no substantive suggestions for improvement (19 (90.5%)).

## Discussion

Engine-driven NiTi instruments improve the quality of root canal treatments performed by undergraduate students, particularly by enhancing filling quality and reducing ledge formation without increasing other iatrogenic risks. Nevertheless, these conclusions are based on evidence of low certainty [10].

Mechanical instrumentation performed by undergraduate dental students with no prior experience was faster and had reduced apical deviation [14]. This highlights the potential for the inclusion of mechanical instrumentation in undergraduate dental curricula.

All participating students had prior experience with mechanical preparation systems and were intentionally selected to ensure familiarity with rotary instrumentation. This approach aimed to isolate the novelty of the tested techniques rather than the concept of engine-driven files, allowing a more objective, experience-based comparison. The inclusion of students who had completed two clinical semesters further supported this objective, as they are less likely to have developed strong procedural preferences or methodological bias compared to more experienced clinicians, who tend to rely on their previous clinical experience when making decisions regarding treatment approaches [15,16].

To minimize response bias, students were informed that the investigator had no preference for either technique and were encouraged to provide unbiased evaluations based solely on their experience. In addition, a structured preparatory session was conducted, providing a comprehensive overview of canal ledge formation, its clinical implications, and various management strategies. This ensured a consistent level of background knowledge and likely contributed to a more informed and meaningful evaluation of the techniques.

Students’ choices regarding ledge management did not differ significantly based on prior experience. However, an important observation was that none of the students without previous exposure to ledge formation selected the option of leaving the ledge unbypassed, whereas this option was chosen only by a small proportion of experienced students. This pattern may reflect differences in clinical perception shaped by prior exposure, whereby students with previous experience may have greater awareness of the practical challenges and limitations associated with ledge bypassing and may therefore consider non-intervention a pragmatic option in certain cases. This interpretation is consistent with the findings of Veronica et al. (2026), who reported that practitioners’ professional experience was associated with differences in their awareness of the causes of ledge formation and in some aspects of ledge management [17]. In contrast, students without previous exposure may have more idealized expectations regarding the feasibility of successful ledge management. Nevertheless, given that the overall association between prior experience and students’ choices was not statistically significant, these observations should be interpreted as a potential trend rather than evidence of a definitive effect of clinical experience on decision-making.

Students perceived both techniques as comparable in terms of learning and application difficulty, with no significant differences observed either between the techniques or in the need for supervisor assistance. These findings suggest that both procedures imposed a similar level of technical and cognitive demand, without additional complexity or increased reliance on supervision. This may reflect the adequacy of prior instruction and the comparable procedural requirements of both methods.

The T-Mode technique was significantly faster than the manual insertion technique, with an average reduction of approximately 114 seconds (~1.9 minutes), demonstrating both statistical and clinical relevance with a moderate effect size. This improvement was further supported by consistently lower mean and median times, reflecting better procedural efficiency and overall consistency. From a clinical perspective, such time savings may become more relevant when accumulated across multiple procedures, potentially enhancing workflow efficiency. Given the within-subject design, where each participant performed both techniques, inter-operator variability was inherently controlled. The nearly identical standard deviations further confirm that the dispersion of performance remained consistent across techniques. Therefore, the observed reduction in mean time is more likely technique-related rather than operator-dependent.

The objective time analysis demonstrated a clear performance advantage for the T-Mode technique, which was significantly faster than the manual insertion technique. At the group level, students were generally able to identify this overall trend; however, this recognition did not consistently translate into accurate individual-level judgments, as agreement with actual performance remained weak, indicating a discrepancy between objective performance and subjective perception.

This gap may be attributed to perceptual and cognitive factors, including difficulty in accurately estimating time differences during clinical tasks, cognitive bias, and a tendency to favor newer or more technologically advanced techniques. Additionally, perceived ease of use and workflow smoothness may have led to an overestimation of speed, with more intuitive techniques being judged as faster regardless of actual performance.

Overall, these findings indicate that subjective perception of efficiency is influenced not only by actual performance but also by cognitive interpretation and perceptual limitations. Accordingly, objective performance gains are not always fully reflected in subjective assessments, as students may have limited ability to critically evaluate performance differences [18,19]. This is consistent with previous reports of a weak correlation between objective and subjective evaluation in procedural performance [20,21], highlighting the importance of integrating both objective and perceptual assessments in the evaluation and teaching of clinical techniques.

Students showed a general tendency to favor the T-Mode technique in terms of both overall preference and perceived quality of results; however, these differences were not statistically significant, indicating a comparable subjective evaluation between the two techniques. This may reflect the limited clinical experience of the participants, as both techniques are likely to yield satisfactory outcomes when properly performed, and at this stage of training, students may have limited ability to critically differentiate subtle variations in procedural outcomes, particularly in an experimental setting that does not fully replicate real clinical conditions. This experimental context may further limit their capacity to anticipate practical challenges and fully appreciate how clinical constraints could influence the application of each technique in real-world practice.

Hands-on training and experimental learning methods have been shown to enhance learning outcomes significantly [22,23]. In the present study, students reported that participation in the experiment provided clear educational value, reflecting the importance of practical exposure in understanding and managing canal ledge complications in endodontics. Most participants were unfamiliar with one or both techniques prior to the study and subsequently expressed willingness to adopt them in future clinical practice. This suggests that early exposure to new techniques not only improves theoretical and practical understanding but may also positively influence future clinical decision-making.

Students reported that the pre-instruction was adequate, reflecting the effectiveness of the structured theoretical preparation and individualized practical training provided before the experiment. The instructional design included a comprehensive explanation of canal ledge formation, its clinical implications, and an organized framework that allowed opportunities for clarification and one-to-one guidance. This likely contributed to students’ ability to perform the procedures appropriately and evaluate both techniques objectively.

Overall, students reported a positive and balanced experience with both techniques. The manual insertion approach was mainly associated with technical difficulty during file attachment but valued for its tactile feedback and safety, whereas the T-Mode technique was more frequently described as faster, safer, and more acceptable. Some students also preferred a combined, case-dependent approach, based on anatomical considerations, technical challenges, and equipment availability, reflecting flexible clinical reasoning. Satisfaction was generally high, with minimal suggestions for improvement, indicating good acceptance of the instructional design and both techniques. A general tendency toward integrating one or both techniques in clinical practice was observed.

Students demonstrated a clear tendency to incorporate the T-Mode technique into future clinical practice, either alone or in combination with other approaches. This preference likely reflects familiarity, perceived ease, and a positive learning experience among less experienced operators. Early exposure to new techniques during undergraduate training appears to influence clinical decision-making and the selection of treatment approaches in future practice [24]. In addition, the structured step-by-step instructional approach may have facilitated skill acquisition and contributed to the positive acceptance of the technique [25]. Overall, these findings highlight the role of early training in shaping future clinical behavior and supporting informed decision-making.

This study extends beyond educational outcomes by providing insight into how structured exposure to clinical techniques influences the relationship between technical performance, cognitive evaluation, and behavioral response in early-stage clinicians. The integration of objective performance data with subjective perception and future intention highlights a measurable gap between actual and perceived efficiency. This discrepancy underscores the importance of incorporating both objective and perceptual assessment methods in clinical training to achieve a more comprehensive evaluation of procedural performance. Collectively, these findings support the early introduction of contemporary techniques within undergraduate curricula and contribute to a more balanced, evidence-based approach to technique evaluation, which may inform future educational strategies and research in endodontics.

However, a few limitations should be considered when interpreting the findings of this study. The relatively small sample size may limit the generalizability of the results, while the use of simulated resin blocks, although allowing for a standardized comparison, may not fully reflect the anatomical complexity and clinical conditions of natural root canals. Furthermore, the fixed order in which the two techniques were performed may have introduced a potential learning or order effect. The evaluation focused primarily on procedural time and students’ perceptions and did not include an objective assessment of ledge correction quality or clinical safety. Further studies using larger samples and clinically relevant models, with objective assessments of procedural outcomes, would help build on these findings.

## Conclusions

Within the limitations of this study, the T-Mode technique was associated with significantly shorter procedural times than the manual technique among undergraduate students, while both techniques received comparable ratings in terms of perceived difficulty and overall evaluation. The findings also revealed a discrepancy between objective procedural time and students’ subjective perceptions of efficiency. These results highlight the value of combining objective performance measures with subjective assessments when evaluating endodontic techniques in undergraduate education. Early exposure to different endodontic techniques during undergraduate training may contribute to shaping future clinical preferences and decision-making.

Overall, the findings highlight the value of structured training and early exposure to contemporary techniques in undergraduate endodontic education. However, further studies incorporating larger samples and objective assessments of procedural quality and safety are needed before concluding the clinical effectiveness or safety of either technique.

## Figures and Tables

**Figure 1:**
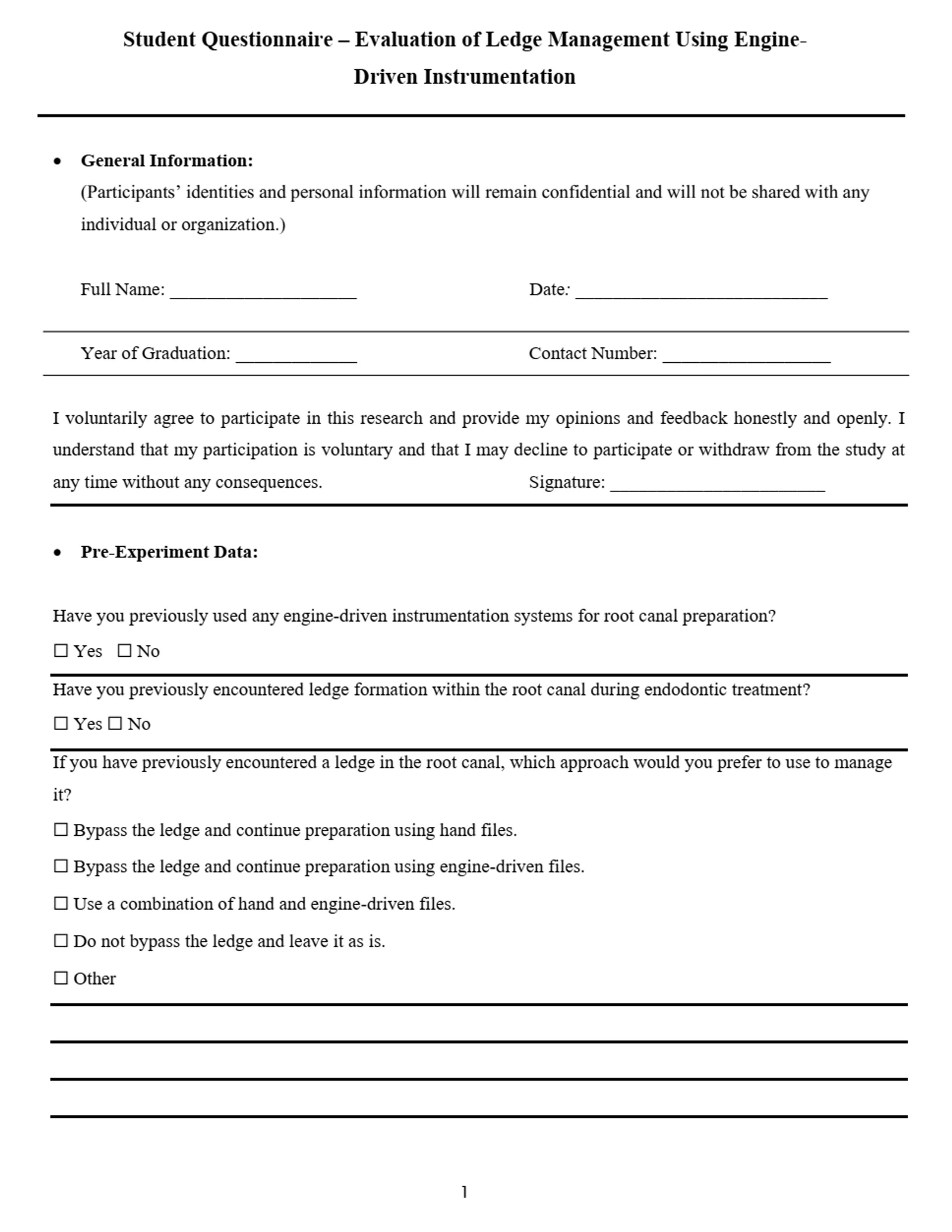
General Information and pre-experiment data section of the questionnaire

**Figure 2:**
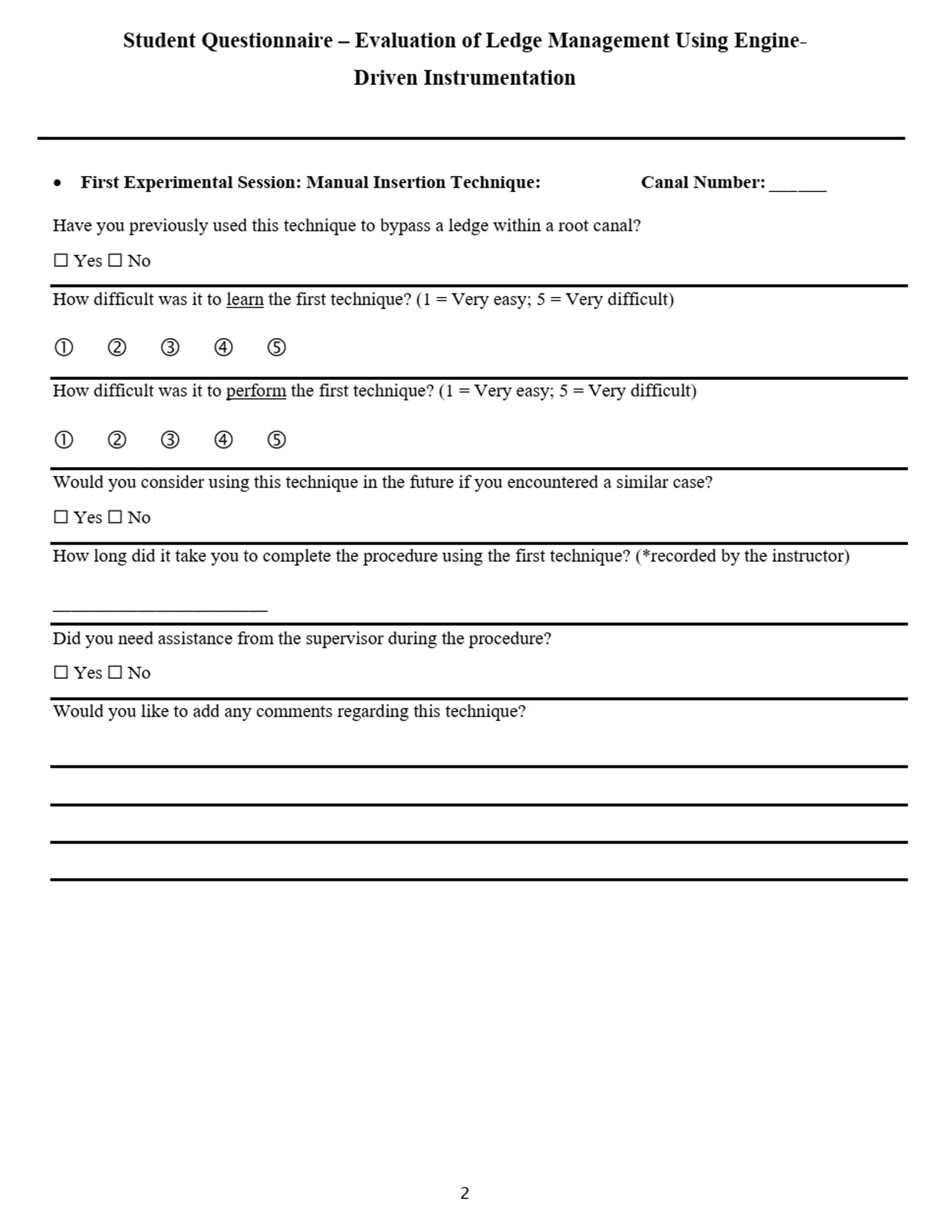
First experiment section of the questionnaire

**Figure 3:**
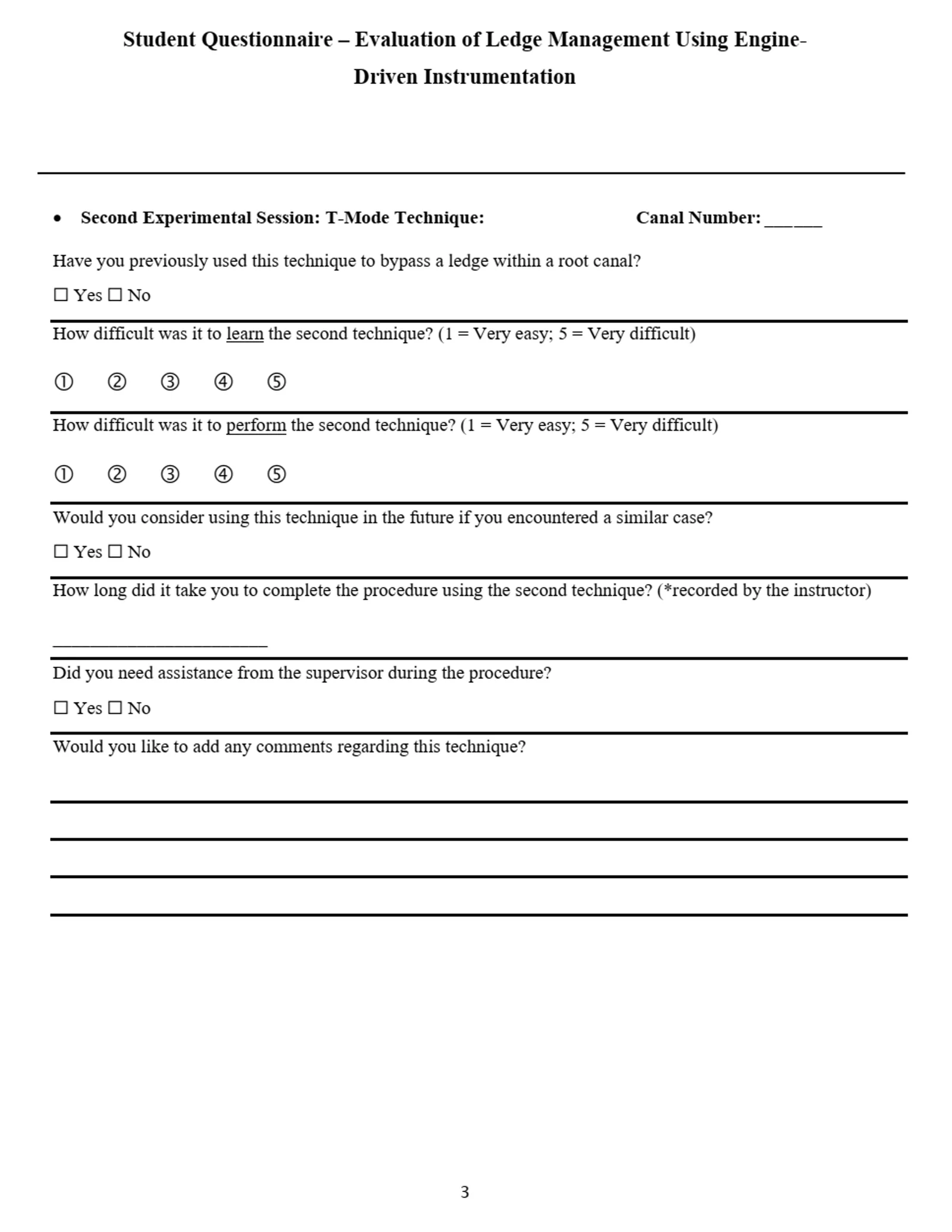
Second experiment section of the questionnaire

**Figure 4:**
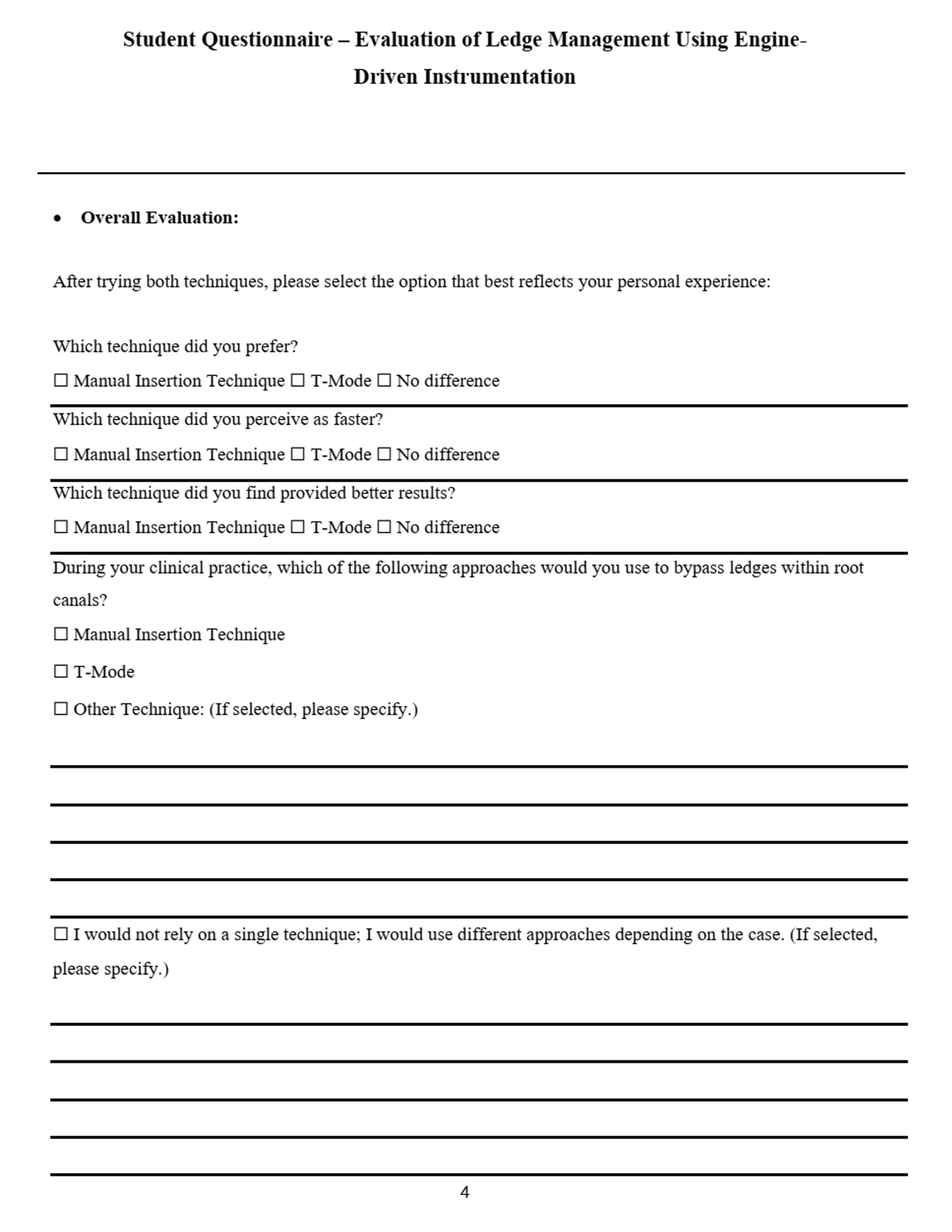
Overall comparative evaluation section of the questionnaire

**Figure 5:**
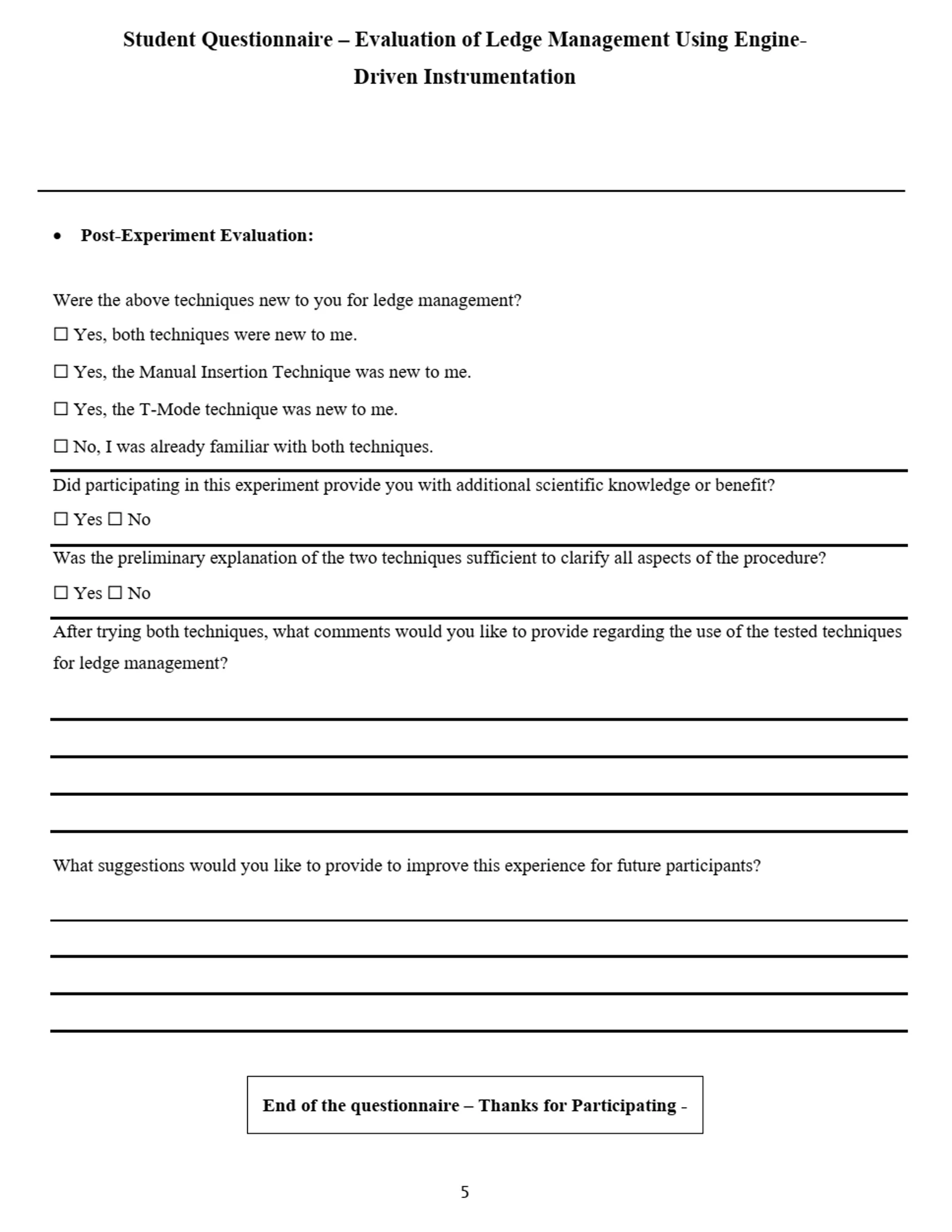
Post-experiment evaluation section of the questionnaire

## Appendices

Appendix A

Figure 1 presents the questionnaire on general information and information regarding any prior experience with ledge management.

Appendix B

Figure 2 presents the questionnaire used to document the initial experience of the students with the technique.

Appendix C

The questionnaire used for the second experimental session is presented in Figure 3.

Appendix D

The overall comparative evaluation section of the questionnaire is shown in Figure 4.

Appendix E

The post-experiment evaluation section of the questionnaire is presented in Figure 5.
